# Advantages of run-reverse motility pattern of bacteria for tracking light and small food sources in dynamic fluid environments

**DOI:** 10.1098/rsif.2025.0037

**Published:** 2025-06-18

**Authors:** Ksenia Guseva, Ulrike Feudel

**Affiliations:** ^1^ Centre for Microbiology and Environmental Systems Science, University of Vienna, Wien, Austria; ^2^ Carl von Ossietzky University of Oldenburg, Oldenburg, Germany

**Keywords:** marine bacteria, chemotaxis, microswimmers, turbulence, motility patterns

## Abstract

Marine bacteria are fundamental to the processes and cycles that sustain ocean ecosystems. Their activity at small scales, where they search for food sources in a highly heterogeneous and dynamic environment, for example controls the decomposition of organic matter. To be effective, these microorganisms have evolved sophisticated behaviours, which include extremely rapid swimming speeds, a precise chemosensing ability and particular swimming patterns. One of these peculiar motility patterns often recorded in the ocean is run-reverse (Mitchell *et al* 1996 Clustering of marine bacteria in seawater enrichments. *Appl. Environ. Microbiol.* **62**, 3716–3721. (doi:10.1128/aem.62.10.3716-3721.1996), Stocker R. 2011 Reverse and flick: hybrid locomotion in bacteria. *Proc. Natl Acad. Sci. USA* **108**, 2635–2636. (doi:10.1073/pnas.1019199108), where bacteria alternate between forward (pushing) and backwards (pulling) swimming modes. In this study, we investigate whether this swimming pattern offers advantages to microorganisms that actively track small and light food sources carried by a dynamic flow. For that we develop an individual-based model, where elongated self-propelled particles (microswimmers) track passive food particles (tracers) in a random kinematic flow field, also known as synthetic turbulent flow. We compare the widely studied motility pattern of run-and-tumble with the run-reverse mode used by marine bacteria. Our results reveal a significant hydrodynamic advantage of the run-reverse motility pattern of bacteria combined with their elongated shapes for efficiently tracking light food sources in dynamic fluid environments.

## 1. Introduction

Marine environments at the small scale are very heterogeneous and dynamic, characterized by ephemeral nutrient availability [1]. To be able to quickly detect and move towards food sources represents an important fitness advantage in this type of environment. To thrive in these complex habitats, marine heterotrophic bacteria have evolved specialized behavioural strategies at the single-cell level, including high swimming speeds, chemotaxis and special motility patterns (e.g. run-reverse mode). The foraging strategies that result from these behaviours play a crucial role in shaping the microscale spatial distribution of these microorganisms, which has potentially significant implications for ecological and biogeochemical processes at larger scales [2,3]. Despite numerous studies of the efficiency of chemotactic ability of marine microorganisms [4], the mechanisms underlying their foraging success in turbulent environments remain poorly understood. In this work, we analyse the advantages of swimming strategies of marine bacteria to track small food sources (ranging from 1 to 100 μm) in a dynamic fluid flow. For that, we build an individual-based model that consists of microswimmers, i.e. self-propelled particles, which track passively advected food sources in a synthetic turbulent flow.

Although microbes impact processes and cycles at the global level, they live and interact with their environment at the microscale characterized by moving resource hotspots and fine-scale chemical gradients. Among different food sources that heterotrophic bacteria exploit in the ocean are microscale ephemeral organic substances and patches released by phytoplankton or created by cell lysis, as well as larger particles such as marine aggregates, which are hotspost of microbial activity [5,6]. In this study, we are interested in the bacterial foraging for small food sources (e.g. phytoplankton cells, other dead microbes and polysaccharide gels, which are of the order of few micrometres or even smaller). They are passively advected by the flow field and constitute hotspots containing oligosaccharides, amino acids and essential nutrients. Because of the significant advantage of locating and exploiting these hotspots, many marine bacteria have evolved to effectively respond to certain chemical stimuli, adopting chemotactic foraging strategies [7–10]. In addition they developed very fast motility, with an average swimming speed of around 100 μm s^−1^ [11] (speeds vary in the range 1–1000 μm s^−1^ for bacteria, with the upper limit given for *Ovobacter propellens* [4,12]). Despite being energetically very costly, this motility confers a good trade-off to the organism, as estimated by Taylor *et al*. [13] for the case of bacterial consumption of thin filaments of dissolved organic matter spread by a turbulent flow. Another peculiarity of marine bacteria is that they—in their vast majority (90%)—have a single flagellum [14], and because of that adopt particular motility patterns. It is useful to compare their motility with the most well-studied bacteria *Escherichia coli*, a gut bacterium with several flagella, which swims in straight runs with random reorientation events of about $70^{\text{o}}$ [15]. In contrast to the typical enteric bacteria, such as *E. coli*, marine bacteria due to their single flagellum have another reorientation dynamics often moving using run-reverse (reorientation by $180^{\text{o}}$) or sometimes run-reverse-flick, with reorientation by $180^{\text{o}}$ and $90^{\text{o}}$ [16,17].

All of the strategies described above may confer advantages to the peculiar microenvironment inhabited by marine bacteria, where these bacteria are constantly subjected to changing physical forces, imposing constraints on their locomotion and orientation. At a small scale fluid flow spins and re-orients microorganisms, which may interfere with their ability to track food gradients [18]. Therfore, the interplay between microbial motility and hydrodynamics is particularly significant to understand their ecological success. In this context, the run-reverse motility pattern has been proposed as an adaptation that enhances the chemotactic abilities of marine bacteria, allowing them to effectively track and remain near microscale food patches, especially under turbulent conditions [19,20]. However, due to the difficulty of observing bacterial tracking behaviour *in situ*, our current understanding of the process is primarily derived from numerical simulations and microfluidics experiments, which often involve considerably simplified systems. Research in this area can be categorized into two main lines of investigation, concerned with: (i) the effect of the fluid flow on swimming trajectories [21,22], reorientation dynamics and spatial organization [18,23] of non-chemotactic swimming bacteria (microswimmers) and (ii) the interplay between motility patterns and chemotaxis in very simplified flow scenarios [11,24,25]. In particular, the work of Rusconi *et al.* [18] demonstrated the negative effects the flow field can have on chemotactic response and foraging of bacteria. While these studies have contributed to our understanding of the potential adaptations of microbial swimming patterns, there remains a notable gap in research concerning the foraging dynamics in ocean-like turbulent environments. In this study, our objective is to bridge this gap and comprehensively address the role of motility patterns in dynamic fluid flows, taking into account the interplay between the flow, swimming, reorientation (motility mode) and chemotaxis. Our study addresses the follwing question: How do the swimming patterns affect the efficiency in foraging for small organic food sources advected by a dynamic flow field? In other words, would run-reverse motility pattern, common to marine microorganisms, offer any advantage in a turbulent environment?

We approach this problem by first investigating the effect of motility patterns on the spatial distribution of microswimmers in random flows in the absence of food sources. We show that elongated microswimmers without active reorientation exhibit a heterogeneous distribution in space. When random reorientation events are taken into account, this heterogeneity persists only for elongated swimmers with run-reverse motility pattern. Next, we analyse the consequence of motility patterns in the tracking behaviour of microswimmers when the food sources are introduced. Our simulations show a strong advantage of swimmers with run-reverse motility pattern and elongated shapes.

The paper is organized as follows. First, in §2, we describe the implementation of all components of the individual-based model of chemotactic microswimmers in a flow field. Next we present the results, which are divided into two parts. In the first part, we analyse the effect of the motility patterns on the spatial distribution of microswimmers in the absence of food sources (§3.1). In the second part, we describe how the motility patterns influence the chemotactic response and tracking of passively advected food particles (§3.2). We conclude in §4.

## 2. Model

Here, we introduce all the necessary components of our model: first, we describe the equations used to model the motion of elliptical and spherical swimmers respectively advected by a two-dimensional random flow field; second, we explain the implementation of the passive advection of the food source and the implementation of chemotaxis.

### 2.1. Elongated microswimmers

**Figure 1.**
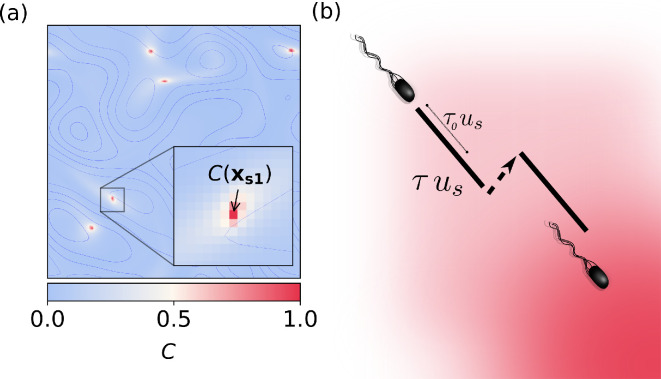
(a) A snapshot of the spatial distribution of the food (chemoattractant) concentration *C*; we represent five food particles, with coordinates $x_{si}$ with i∈{1...5}, considering κ=0.01. We use countour lines (blue) to depict the flow field (u). (b) The trajectory followed by a bacterium in a food concentration; notice the extended runs ($\tau u_{\text{s}}$) towards the food source (bold lines) and the contraction of the runs away from the food source (dashed line). For comparison, see $\tau_{0}u_{\text{s}}$ which represents the extent of the run in homogeneous conditions (no chemoattractant).

We use an individual-based model where the motion of each bacterium is described individually. As a simplification, we assume that the background flow u is not affected by the swimming of bacteria. The flow u(x,t) is two dimensional and is constructed to mimic properties of homogeneous, isotropic turbulence at the dissipative scale. For this reason, it predominantly consists of vortices of approximately fixed size, $\lambda_{f}$, which are randomly distributed within the observation region (with dimensions *L* × *L*, *L* denoting the length of each side of the region). These vortices rotate with a mean absolute velocity $u_{f}$. At the same time each one of the vortices fluctuates, deforms and shrinks and eventually disappears after a time $\tau_{f}$, replaced by new growing vortices (see a snapshot of the velocity field in figure 1a). Here we can establish two connected time scales: $t_{f}=\lambda_{f}/u_{f}$ (or similarly the time a flow element takes to circle around a vortex $t_{f}^{\prime }=2\pi \lambda_{f}/u_{f}$), and the dissipation time $\tau_{f}$. We parametrize the flow in such a way to guarantee that the Kubo number, defined as $Ku=\tau_{f}/t_{f}$ would not be very large to ensure a turbulent scenario (it is estimated to be of the order of unity according to [26]). The details of the flow implementation can be found in the electronic supplementary material and in [27–29]. Note that for a realistic scenario, the length and time scales are given in seconds and millimetres considering that the dissipation of turbulent kinetic energy of the top layer of the open ocean is found in the interval 10^−9^–10^−6^ m${,}^{2}$ s^−^${,}^{3}$ [30] (details in electronic supplementary material).

Each bacterium is modelled as an ellipsoidal object, small enough to ignore any inertial effects. We consider them as active self-propelled particles (microswimmers), which are not only advected by the flow field u but are characterized by an intrinsic swimming velocity $u_{s}$. In other words, the total velocity of a bacterium is given by a vector sum of the fluid velocity at its position and the swimming velocity. While the magnitude of the swimming velocity $u_{s}$ is assumed to be fixed, its direction p changes by two different mechanisms, in a continuous and discontinuous way. The first mechanism assumes that the direction p changes through the action of the flow on the bacterium, this rotation depending on the shape of the organism. The second type of rotation is discontinuous and is performed by the bacterium itself. It consists of a sudden reorientation that might also be influenced by external factors such as chemotaxis. In summary, we implement (i) periods of persistent swimming with smooth changes of direction due to the properties of the flow, interrupted by (ii) instantaneous abrupt turns or ‘tumbles’.

During the intervals of persistent swimming (‘runs’, (i)), the motion of bacteria can be described by [31]


(2.1) $$\frac{\text{d}r}{\text{d}t}=u+u_{\text{s}}p,$$

where the unit vector p is given by


(2.2) $$\frac{\text{d}p}{\text{d}t}=\frac{1}{2}\omega \times p+\alpha p\cdot E\cdot (I-pp),$$

where ω=∇×u is the vorticity and E is the rate of strain tensor of the flow. The first term of equation (2.2) represents the change of the swimming direction by a coupling to the flow vorticity and the second one is due to a coupling to the rate of strain. The influence of the particle shape on realignment is given by the parameter α, which is defined as $\alpha =r^{2}-1$, where r is the ratio between the major and minor axis of an ellipsoid (giving therefore particle eccentricity). Elongated particles characterized by α>0 are rotated by the action of the second term in equation (2.2). On the other hand for spherical particles α=0, and hence the second term in equation (2.2) vanishes.

As mentioned previously, the persistent swimming is interrupted from time to time by sudden random changes of swimming direction, p=[cos⁡(θ(t)),sin⁡(θ(t))] (ii). This event is instantaneous and consists of a rotation by a fixed angle $\theta_{\text{m}}$ with some small additional normally distributed noise σ. The new direction of motion becomes


(2.3) $$p=\left[\cos(\theta (t)+\theta_{\text{m}}+\sigma ),\sin(\theta (t)+\theta_{\text{m}}+\sigma )\right].$$

The angle $\theta_{\text{m}}$ is selected from a probability density $g(\theta_{\text{m}})=1/2\left(\delta (\theta_{\text{m}}-\theta_{\text{m0}})+\delta (\theta_{\text{m}}+\theta_{\text{m0}})\right)$. For the three motility patterns we have run-and-tumble ($\theta_{\text{m0}}=70^{\text{o}}$), run-reverse ($\theta_{\text{m0}}=180^{\text{o}}$) [24]. Small Gaussian noise σ is also added to the rotation angle ($\sigma ∼N(0,5^{\text{o}})$).

After this instantaneous rotation, the dynamics returns to equations (2.1) and (2.2). The time interval between these direction changes τ, in the condition of a homogeneous environment, has a fixed average value $\tau_{0}$ (see table 1). We implement the randomness of this process by assuming a probability of changing direction in each time step Δt


(2.4) $$\begin{array}{lcr} & P_{t}=\tau /\Delta t. & \end{array}$$

**Table 1.** Parameters used for chemotaxis.

symbol	parameter	value
$\alpha_{c}$	characteristic timescale	300 s
$K_{d}/C_{0}$	chemoattractant availability	1
$\tau_{0}$	mean swimming time in homogeneous environment	1 s

In this way, the ‘run-and tumble’ (‘run-reverse’) motion of bacteria is modelled as a simple Poisson process, where all the tumbling (reversal) events occur completely at random but with predefined average τ (in homogeneous environment $\tau_{0}$). Importantly, we assume that the background flow field affects neither the swimming behaviour nor the tumbling direction chosen by the bacterium and the angle of rotation $\theta_{m}$ depends only on the motility pattern. Finally, we assume that all compared microorganisms spend the same amount of energy to propell themselves in the flow, independent on the motility pattern. We also consider that through the timelapse of the simulation all of the bacteria are in constant active motion tracking the advected food sources.

### 2.2. Food sources and chemotaxis

To study the question of the impact of the swimming strategy on the success of finding food sources in a turbulent environment, we introduce five randomly placed food particles, which may represent phytoplankton, particulate organic matter or even marine aggregates passively advected by the flow field. Since these sources are composed mainly of organic material we consider them to be very light (excess density $\Delta \rho =\rho_{\text{f}}-\rho_{\text{p}}∼0.01$ g cm^−^${,}^{3}$ [32], $\rho_{\text{f}}$ and $\rho_{\text{p}}$ indicating fluid and particle densities, respectively) and small (1–100 μm). This leads to a small Stokes number St≪1 and therefore these particles would move as tracers, following exactly the trajectories of the flow [32]. In other words, the position $x_{si}$ of the ith source evolves according to $\text{d}x_{\text{si}}/\text{d}t=u(x_{\text{si}})$. Though these food sources are very small and we approximate them by point-like sources moving in space, they also release chemoattractants (e.g. small sugars and amino acids ) into their surroundings. This chemoattractants C are advected by the surrounding flow field and decay everywhere except at $x_{si}$ with a rate κ


(2.5) $$\begin{array}{r} \frac{\partial C}{\partial t}+u\cdot \nabla C=\left\{ \begin{array}{ll} 0, & \text{if }x=x_{si},i\in \{1...n\}\\ -\kappa C, & \text{otherwise}, \end{array}\right. \end{array}$$

where n is the number of food sources. Note that we approximate the $C(x_{si})=C_{0}$ to be a fixed value for each source (in other words, we assume that during the time window of our observation its change is insignificant). The spatial distribution obtained after a short transient is shown in figure 1a.

As we have described in the previous subsection, the forward swimming motion of bacteria is interrupted by occasional random changes in swimming direction. Organisms search for food along a gradient of chemoattractants released by the food source indicating its location in space. This behaviour called chemotaxis is modelled by considering that the time intervals, τ, between random changes of swimming direction are no longer constant but depend on the concentration gradient that a bacterium experiences along its path [33,34]. We use the state-of-the-art modelling approach for chemotaxis, developed from observation of *E. coli* [33]. The main idea of the model is that the background food concentration affects the running times τ and consequently the probability of changing the swimming direction $P_{t}$; see equation (2.4). More precisely, a bacterium experiencing an increasing concentration of food during a run has a smaller probability $P_{t}$ of changing its swimming direction. On the contrary, if it senses a decrease of concentration, $P_{t}$ increases. To describe this effect, it is considered that the expansion and contraction of running times, τ, is proportional to the rate of change in the number of active receptors on the bacterial cell membrane. The chemotactic receptors get active when they bind to a chemoattractant, i.e. a food source such as sugar or amino acids. We also assume that the food concentration is large enough not to be affected by the binding dynamics. The fraction of activated receptors, $R_{\text{b}}$, changes depending on the gradient along the swimming trajectory of bacteria [33], and therefore we assume that it is a function of the material derivative $\text{D}C/\text{D}t=\partial C/\partial t+{\text{u}}_{s}\cdot \nabla C$ as proposed by Rivero *et al.* [35] and following a series of works on chemotaxis (see [36–38]). In other words, the change in the fraction of activated receptors $\text{d}R_{b}/\text{d}t$ depends on the rate of change of the concentration of the chemoattractant as seen by a bacterium along its trajectory, which is given by DC/Dt (i.e. $\text{d}R_{b}/\text{d}t\propto \text{D}C/\text{D}t$; notice that when bacteria do not swim we have $\text{d}R_{b}/\text{d}t\propto \partial C/\partial t$). However, the fraction of activated receptors also depends on its association or dissociation time with new molecules. In addition, we are accounting for the fact that there is a finite number of receptors on the cell wall. This association–dissociation dynamics determines the half saturation constant $K_{d}$ of a given receptor, which indicates the concentration of chemoattractant where half of the receptors are activated. Taking into account these assumptions, the change of the fraction from the total number of receptors bound $R_{b}$ to the chemoattractan (e.g. organic molecule) is modelled with the following relation:


(2.6) $$\frac{\text{d}R_{b}}{\text{d}t}=\frac{K_{d}}{(K_{d}+C{)}^{2}}\frac{\text{D}C}{\text{D}t}.$$

The running time for a bacterium, therefore, is a dynamic quantity that changes according to the concentration gradient experienced by a bacterium, according to


(2.7) $$\tau =\tau_{0}e^{\alpha_{c}\frac{dR_{b}}{dt}},$$

where $\tau_{0}$ is the mean time for swimming in the absence of a food gradient (see §2.1) and $\alpha_{c}$ is an amplification factor. The values of parameters used in the simulations are given in table 1. Notice that the runs are extended when a bacterium senses an increase in concentration along its trajectory (DC/Dt>0) and contracted when it records a decrease (DC/Dt<0); see right panel of figure 1. Due to the stochastic nature of bacterial motion, we use τ to compute a probability of change of direction in an given instant. In this way for each time step Δt, each bacterium has a probability to switch its swimming direction randomly which is given by equation (2.4).

In all the following analysis, we measure the time and length in the flow characteristic units, $t_{f}=u_{f}/\lambda_{f}$ and $\lambda_{f}$, respectively. The non-dimensional values of the parameters are set to $u_{f}^{\prime }=1$, $\lambda_{f}^{\prime }=1$, $\tau_{f}^{\prime }=2\pi$, $L^{\prime }=2\pi$. We vary the velocity speed of the bacteria $u_{s}^{\prime }=u_{s}/u_{f}\in \left[0,4\right]$ and the time between changes in directions $\tau_{o}/t_{f}\in \left[0,12\right]$ (testing also the conditions when bacteria extend their standard running time beyond 1 s). The code for the simulation reported here can be found in the Github repository: https://github.com/kseniaguseva/Swimmers [39].

## 3. Results

### 3.1. Microswimmers in an homogeneous environment

To start, we investigate how the different motility patterns influence the spatial distribution and the dynamics of elongated microswimmers in the absence of a food gradient. We assume periodic boundary conditions and set the domain size to $L=2\pi \lambda_{f}$. Here we differentiate three types of swimmers: (i) *simple swimmers* (no tumbling or reversing motility), i.e. τ→∞; (ii) *run-and-tumbling swimmers* ($\tau =\tau_{0}$ with execution of a turn with $\theta_{m}=70^{\text{o}}$) and (iii) *run-reversing swimmers* (same as the previous case but with a turn of $\theta_{m}=180^{\text{o}}$). The objective is to compare the spatial distribution and alignment of microswimmers with these three motility types with the flow field.

**Figure 2.**
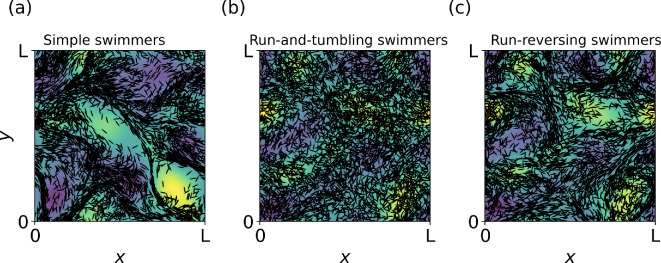
Spatial distribution of elongated (α=0.98) microswimmers at $60t_{f}$ ($u_{s}=0.5u_{f}$) with (a) simple swimmers, (b) run-and-tumbling swimmers and (c) run-reversing swimmers. (b,c) We consider that switching swimming directions is $\tau_{0}=t_{f}$. The background colour gradient represents the vorticity of the flow field.

**Figure 3.**
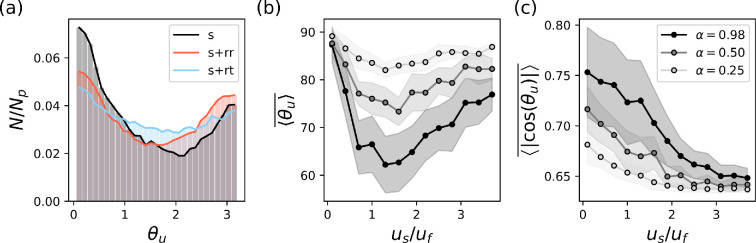
(a) Fraction of active swimmers ($N_{p}=5\times 10^{4}$) with a given angle $\theta_{u}$ between their swimming direction p and the flow velocity u, for different swimming patterns: s—simple swimmers; s+rr—run-reversing swimmers; and s+rt—run-and-tumbling swimmers (snapshot after $60t_{f}$). (b,c) The alignment between simple swimmer's swimming velocity and the flow field for different values of swimming speed ($u_{s}/u_{f}$) and shape (α) of microswimmers. The alignment is represented by (b) ⟨θu⟩ or (c) $\bar{\left\langle |\text{cos}(\theta_{u})|\right\rangle }$. Both quantities constitute averages over several snapshots (one every $0.1t_{f}$, until $50t_{s}$ after eliminating a transient of $2t_{f}$). The shaded area represents the variation of each one of these averages around the mean.

It is known that passive particles of elongated shape show a particular alignment with the flow structures [40,41]. For active elongated particles, the overall dynamics and alignment lead to heterogeneous spatial distributions including the possibility of the formation of transport barriers [22]. Although the clustering is quite pronounced in cellular flows [42], it was shown to be weaker in three-dimensional turbulence [43]. In our random flow, the elongated simple swimmers (i.e. with α=0.98) are in agreement with the above listed studies. They demonstrate an alignment with the flow field and concentrate in certain areas, as shown by the snapshot of their spatial distribution in figure 2a. As expected, the random changes in the swimming direction work as perturbations. Moreover, the angle $\theta_{m}$ of the motility pattern determines the scope of loss of this alignment by such perturbations, while for reversing swimmers the non-homogeneous spatial distribution is still observable (figure 2c), it noticeably decreases for run-and-tumble motility (figure 2b). To quantify the effect above, we systematically analyse the alignment between the swimming direction p and the flow field u by the angle between them ($\theta_{\text{u}}$). In all three cases, the histograms of $\theta_{u}$ have u-like shapes, characterized by two maxima: one at $0^{\text{o}}$ and the other at $180^{\text{o}}$. We observe that a large fraction ($N/N_{\text{p}}$) of simple swimmers have their swimming direction aligned with the flow velocity, maxima at $\theta_{u}=0$ (see the asymmetry in the distribution, the average angle is $76^{\text{o}}$); see figure 3. This fraction is smaller for reversing swimmers (figure 3a), with a more symmetric distribution (with average angle $87^{\text{o}}$, which is very close to $90^{\text{o}}$ expected if the distribution would be perfectly symmetric) showing alignment of the front and backward motion (peaks of $\theta_{u}=0$ and peaks of $\theta_{u}=\pi$). Finally, we observe that the distribution almost flattens for tumbling swimmers (figure 3a), with average also close to $90^{\text{o}}$ in this case.

To quantify the alignment described above, we systematically analyse how it changes with other parameters of the system. To do that, we work with $\left\langle \theta_{u}\right\rangle =1/N_{p}\sum_{i=0}^{N_{p}}|\theta_{u_{i}}|$ and $\left\langle |\cos(\theta_{u})|\right\rangle =1/N_{p}\sum_{i=0}^{N_{p}}|cos(\theta_{u_{i}})|$, both averaged over all particles in the system. Note that the first quantity approaches $90^{\text{o}}$ for perfectly symmetric distribution of alignment angles. While the second quantity approaches 1 for perfect alignment and deliberately does not distinguish between front ($\theta_{u}=0^{\text{o}}$) and back ($\theta_{u}=180^{\text{o}}$) motion of bacteria in this case. This means that alignment is considered the same regardless of whether the particle’s orientation is flipped (see the electronic supplementary material, figure S1 for details). We begin our analysis with simple swimmers, whose alignment depends on two key factors: the ratio between their swimming speed to the flow speed ($u_{s}/u_{f}$) and their shape, defined by the eccentricity parameter α. The strongest alignment occurs when the swimming speed and flow speed are comparable ($u_{s}/u_{f}∼1$; see figure 3b). This is because at this point $\left\langle \theta_{u}\right\rangle$ shows a minimum with $u_{s}/u_{f}$, which reflects the asymmetry in angle distribution and therefore the best alignment. For very fast swimmers, the background flow causes only minor perturbations to their general direction and cannot fully rotate them (figure 3b,c). At the same time, an elongated shape (α closer to 1) enhances alignment. To further understand these results, we also analyse the effect of noise on alignment (see electronic supplementary material, figure S3a, c). As expected, we find that higher frequency and greater variance of the perturbation angle inhibit alignment.

**Figure 4.**
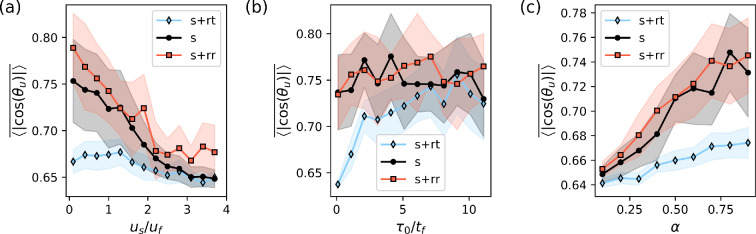
The alignment between the swimming direction p and the flow velocity u for different values of (a) swimming velocity $u_{s}$, (b) average time between switches in swimming direction $\tau_{0}$ and (c) shape of the swimmer α. The different swimming patterns are represented by s—simple swimmers; s+rr – run—reversing swimmers; and s+rt—run-and-tumbling swimmers. The simulations were run with $N_{p}=10^{4}$ swimmers characterized by $u_{s}=0.5u_{f}$, $\tau_{0}=t_{f}$ s, α=0.98, unless otherwise stated. The quantity ⟨|cos⁡(θu)|⟩ represents an average of the alignment taken every $0.1t_{f}$, until $50t_{f}$ (a transient of $2t_{f}$ is discarded). The shaded area represents the variation of each one of these averages around the mean.

Next we analyse how the alignment for the two other types of swimmers differs from that of the simple swimmers described above. The swimming speed $u_{s}/u_{f}$ and eccentricity parameter α have the same effect on these swimmers as well; see figure 4a,b. However, a key factor influencing alignment in this case is the frequency with which microorganisms change direction. As expected, when the directional change frequency is low (large $\tau_{0}$), there is little difference between the motility patterns since the flow has sufficient time to rotate and align the microorganisms. Conversely, when the frequency is high, the alignment of swimmers with run-and-tumble and run-reverse motility patterns diverges significantly. To better understand this, we define a length scale $l_{f}/\lambda_{f}=\tau_{o}\cdot u_{f}/\lambda_{f}$, which represents the distance a swimmer is advected by the flow during the interval $\tau_{0}$. In figure 4b, we see that bacteria get the same alignment as simple swimmers when $l_{f}/\lambda_{f}∼1$. Note that this convergence value will depend on the swimming velocity and the eccentricity of swimmers. In summary here again, we can see a strong difference between reversing and tumbling swimmers: while reversals seem to provide the same alignment as of simple swimmers, shorter intervals between tumbling have the opposite effect; see figure 4b.

### 3.2. Tracking passively advected sources of food: influence of shape and motility pattern

In this section, we analyse the ability of microorganisms to track food by using different swimming strategies. For that we introduce five food sources to the system which are passively advected; for details see §2.2. After the flow field with advected food particles reaches a steady state ($32\;\tau_{f}$), we add chemotactic microswimmers and follow their distribution in space.

**Figure 5.**
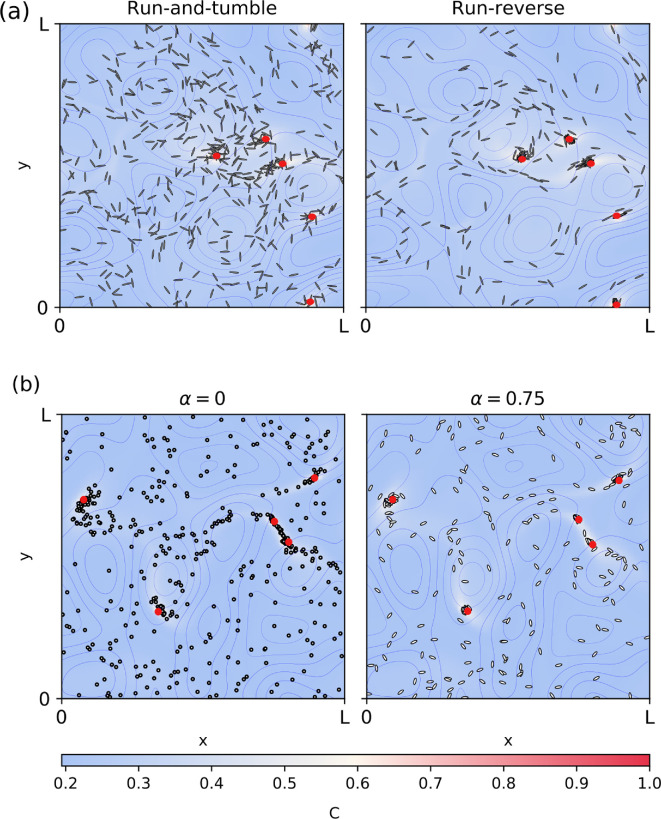
(a) Snapshot at $t=50t_{s}$ of the spatial distribution of 500 microswimmers tracking passively advected sources comparing the two motility patterns (α=0.98). (b) Spatial distribution of 500 microswimmers with run-reverse motility pattern of circular (α=0) and elliptic shapes (α=0.75). We consider swimming velocity $u_{s}=u_{f}$, the parameters of chemotaxis are $\alpha_{ch}=600$ s, $C_{0}/Kd=1$ and $\tau_{0}=t_{f}$. To improve visibility, we marked the position of the food sources as red dots. Note that all plots contain the same number of microswimmers.

**Figure 6.**
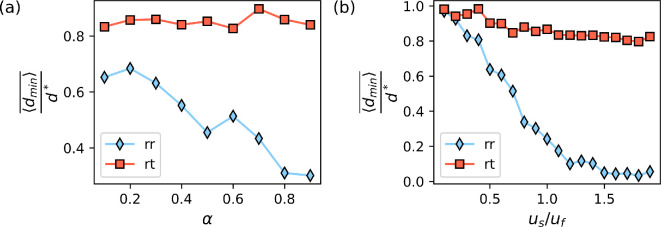
Average distance of microswimmers to the closest food source (a) for different eccentricity parameter α, which changes the shape of the microswimmer, considering $u_{s}=u_{f}$; and (b) for a range of different swimming speeds for elongated microswimmers α=0.75. All values were rescaled to the distances observed in randomly distributed microswimmers (no chemotaxis) $d^{*}=0.25\;\lambda_{f}$. The distance represents a time average over snapshots taken from $t=100t_{s}$ to $t=200t_{s}$.

We start by analysing elongated microswimmers α=0.98, with a swimming velocity comparable to that of the surrounding flow field, $u_{s}=u_{f}$. After a short transient period, most of the microswimmers with run-reverse motility pattern are able to approach and track one of the food sources, while microswimmers with run-and-tumble motility are less successful. We show a snapshot of the spatial distribution of microswimmers at $48\;\tau_{f}$ in figure 5a. To quantify this effect we compute the average distance of microorganisms to their closest food source, given by $\left\langle d\right\rangle =1/N\sum_{i}^{N}(\text{min}(\sqrt{(x_{i}-x_{sj}{)}^{2}+(y_{i}-y_{sj}{)}^{2}}))$, where N is the number of microswimmers, ($x_{i},y_{i}$) is the position of the ith microswimmer and ($x_{sj},y_{sj}$) is the position of the jth source. To offer a reference of how close swimmers are to food particles, we compare the current distance distributions with a fully random scenario, where they are randomly distributed without chemotaxis and swimming. For that we normalize all distance values obtained for our chemotactic swimmers by the mean value of distances $d^{*}$ observed for randomly distributed swimmers, estimated for this case as $d^{*}=0.25\;\lambda_{f}$. For small swimming velocity, as expected, the tracking is not successful and all microswimmers are spread in space by the flow field ($\left\langle d\right\rangle /d^{*}∼1$). For larger velocities swimmers with both motility types show an improvement in their tracking ability, shown by a decrease of the average distance to the source ⟨d⟩. Moreover our simulations show a very strong improvement, with increasing swimming velocity, for a run-reverse motility pattern, while there is only a slight difference for swimmers with run-and-tumble figure 6b.

Finally, we also analyse the effect of the shape on the tracking ability of microswimmers. For run-and-tumble, the shape plays no role and there are no changes in ⟨d⟩ for changes in the eccentricity parameter α. On the contrary, for run-reverse motility pattern the elongated shape gives an advantage; figure 6a. This indicates the importance of the alignment with the flow for the tracking ability of the microswimmers. We show a snapshot of the spatial distribution of circular and elongated microswimmers with run-reverse motility in figure 5b.

In summary, our simulation shows a strong advantage of the run-reverse motility pattern in tracking small passively advected food particles in a two dimensional random flow. Moreover, we have also observed a significant advantage for elongated microswimmers.

## 4. Conclusion

In this work, we have analysed the tracking ability of microswimmers with run-reverse motility pattern in a dynamic fluid, comparing their performance to counterparts with run-and-tumble motility. Our simulation results indicate that despite random switches in swimming direction, microorganisms employing the run-reverse motility exhibit a remarkable ability to align with flow fields, which helps them to efficiently track and follow passively advected food sources. We also show that the shape of the bacteria is another crucial factor that influences alignment and food tracking, and microorganisms with spherical shapes show a disadvantage compared with the elongated ones. These findings provide valuable insights into the possible adaptations that bacteria developed to survive in turbulent flows, enhancing our understanding of the mechanisms behind the efficiency of chemotaxis in complex natural environments.

Previous works have suggested that the interplay between the flow field and bacterial motility may have a significant impact on chemotaxis, particularly in the case of bacteria with elongated shapes. The effect of shear on spatial distribution of elongated bacteria in flows is known from microfluidics experiments [18]. Bacteria swimming in a laminar shear flow exhibit a heterogeneous spatial distribution due to the interplay between the flow and bacterial motility, a phenomenon denoted as shear-induced trapping. This effect is considered to enhance bacterial possible interactions with surfaces [44] and affect chemotaxis. While these studies have analysed the segregation in simple shear flow, our work analyses the swimming behaviour in turbulent-like settings, characterized by the presence of dynamic vortices, that appear, deform and disappear over time. Note that the shape of bacteria and the motility pattern are particularly useful when swimming velocity of bacteria is close in its magnitude to the velocity of the fluid flow (in our analysis in the range $0.2u_{s}$–$2u_{s}$). In the limit of high flow velocities, it is known that tracking becomes ineffective [13] and therefore it would constitute a waste of energy for bacteria. On the other hand in a slow flow field bacteria would be efficient independently of the chosen strategy.

We note that other aspects of bacterial movement, not considered in this study, have the potential to further enhance bacterial tracking efficiency. For instance, certain marine bacteria are capable of regulating their swimming speed, adjusting it to be slower or faster when moving forward or backward [45]. Additionally, some microorganisms exhibit the ability to modulate their swimming speed in response to chemical gradients through a mechanism known as chemokinesis [46,47]. Furthermore, collective effects among microswimmers, including swarming and quorum sensing [48], can significantly impact tracking efficiency as was already shown by Torney *et al*. [49] and they should be taken into account in future investigations.

In this work, we consider relatively small food sources suspended by the flow field. When considering larger particles, such as aggregates (marine snow), in addition to horizontal advection, the vertical movement in the water column as well as deformation of the flow field around such particles have to be taken into account. While small marine snow particles can still be approximated by tracers in the horizontal direction [32,50,51], they would have a settling velocity that also affects their encounter rates with bacteria [52]. In addition, the deformation of the flow field and the hydrodynamic forces near the particle surface were shown to help bacterial colonization in theoretical [37,38] and experimental studies [44].

Our study serves as an initial exploration step into the microbial strategies employed in highly dynamic environments, resembling the conditions bacteria encounter in the ocean. While we have examined the hydrodynamic aspects of the problem, additional factors such as energetics and population dynamics may have shaped the evolution of specific motility patterns in marine bacteria. But these aspects are beyond the scope of this article. Understanding the dynamics of microbial tracking at the small scale within realistic scenarios constitutes a crucial step towards modelling and making prediction on broader processes that characterize ocean biogeochemistry.

## Data Availability

The code for the simulation reported here can be found at Zenodo [39]. Electronic supplementary material is available online [53].
